# Evaluating the Comprehensive Performance of Herbaceous Peonies at low latitudes by the Integration of Long-running Quantitative Observation and Multi-Criteria Decision Making Approach

**DOI:** 10.1038/s41598-019-51425-0

**Published:** 2019-10-21

**Authors:** Jiaping Zhang, Xiaobin Wang, Dong Zhang, Shuai Qiu, Jianfen Wei, Juan Guo, Danqing Li, Yiping Xia

**Affiliations:** 1 Physiology and Molecular Biology Laboratory of Ornamental Plants, Institute of Landscape Architecture, College of Agriculture & Biotechnology, Zhejiang University, Hangzhou, 310058 China; 2 Research & Development Center, Hangzhou Landscaping Incorporated, Hangzhou, 310020 China

**Keywords:** Plant domestication, Abiotic

## Abstract

Enlarging the planting area of economic plants, such as the “Southward Planting of Herbaceous Peony” (*Paeonia lactiflora*. Pall), is significant for improving people’s lives. Peony is globally known as an ornamental because of gorgeous flowers and is mainly cultivated in the temperate regions with relatively cool and dry climates in the Northern Hemisphere. Promoting the landscape application of peony to the lower latitude regions is difficult because of the hot-humid climate. In this study, 29 northern peony cultivars and a unique Chinese southern peony, ‘Hang Baishao’, were introduced to Hangzhou, located in the central subtropics. Annual growth cycles, resistances and dormancy durations were measured, and crossbreeding between the southern and northern peonies was performed for six years, from 2012 to 2017. Based on data collected from the long-running quantitative observation (LQO), a multi-criteria decision making (MCDM) system was established to evaluate the comprehensive planting performance of these 30 cultivars in the central subtropics. ‘Qihua Lushuang’, ‘Hang Baishao’ and ‘Meiju’ were highly recommended, while ‘Zhuguang’ and ‘Qiaoling’ were scarcely recommended for the Hangzhou landscape. This study highlights the dependability and comprehensiveness of integrating the LQO and MCDM approaches for evaluating the introduction performance of ornamental plants.

## Introduction

Enlarging the planting area of a cultivated plant or extending the ecological amplitude of a wild plant with significant economic value is an important endeavor in agriculture, biology, forestry and horticulture^1–6^. The “northward planting of south species”, “southward planting of north species” and “downward planting of alpine species” are three main categories in this research^6–8^. Increasingly, global warming facilitates the first category but makes the latter two categories more difficult to implement^3,9,10^. Nearly all plants in subtropical regions face the threats from warm winters and burning hot summers, and their distribution and cultivation areas have been declined seriously and are gradually moving to higher latitudes. Therefore, in the Northern Hemisphere, the “southward planting of northern-important economic plants” is a greatly valuable measure for maintaining or increasing the plant diversity, ecological balance and people’s living standard in many low-latitude regions (the areas between N 30°00′ to S 30°00′ of the earth). Only through many years of rigorous resource introduction, evaluation and crossbreeding can the northern plants be successfully transported to the lower latitudes^11^, which directly determine the screening of superior resource and breeding the new cultivar in the destination area^4,12^.

Many previous studies formulated the evaluation of plant characters as a multi-criteria decision making (MCDM) problem and adopted various methods to study this problem, such as the gray relational analysis (GRA), analytic hierarchy process (AHP), analytic network process (ANP), multi-attribute utility technique (MAUT), simple multi-attribute rating technique (SMART) and technique for order preference by similarity to ideal solution (TOPSIS)^12–14^. These methods place different emphases on the constraints, preferences and priorities of the decision makers and have multi-hierarchies with multi-criteria, which could be assigned many valuable indices corresponding to the important traits of the target plants^12,14,15^. Therefore, adopting one or more MCDM methods to evaluate comprehensive performance is an important precondition for transporting economic plants to a new area that may not have appropriate climatic and suitable environmental conditions^14,15^.

Herbaceous peony (*Paeonia lactiflora*. Pall) is a world-famous and fascinating perennial ornamental with fabulously gorgeous flowers^16^. This perennial plant, with an overwintering bud dormancy trait, is welcomed as an excellent landscape-, pot- or cut-flower in European, North American and Asian countries^1,17–19^. Peony likes temperate and cool climates, and is naturally distributed or largely cultivated in the Hardiness Zones 3 to 7, and is relatively less cultivated in the Zone 8 of China (Supplementary Fig. S1; website: https://www.ars.usda.gov/ARSUserFiles/50301000/Graphics/Climate_china.pdf)^11,20–24^. It is very difficult to transplant peony to more southern areas of the subtropics and even the tropics in China, such as Zhejiang, Jiangxi, Fujian and Guangdong provinces^11,25^ (Hardiness Zones 9 to 11 in Supplementary Fig. S1). The high temperatures and humid climates in these regions severely impede the “Southward Planting of Herbaceous Peony”, which is a classical subject in horticultural research^7,11,22–28^. The global warming trend and greenhouse effect exacerbate the difficulty of achieving this significant objective^10,29,30^; thus, herbaceous peony landscapes are scarce in the low-latitude provinces, such as Zhejiang (Zones 9 in Supplementary Fig. S1)^7,25^.

However, unexpectedly, the plantations of herbaceous peony were extremely prosperous in Zhejiang in the Chinese Southern Song Dynasty (A.D. 1,127–1,279), especially in Hangzhou, which was the capital city of China in this dynasty. Thereafter, peony plantations declined gradually because of natural and political reasons^11^. Only one peony with a single flower type named as ‘Hang Baishao’, was preserved and then cultivated as a medicinal plant for approximately 1,000 years in central Zhejiang. ‘Hang Baishao’ probably has high adaptability to the unsuitable climate of the low-latitude region^7,25^. Therefore, screening the superior cultivar from the northern herbaceous peony directly and creating excellent new germplasm by crossbreeding the southern ‘Hang Baishao’ with traditional northern cultivars are two main approaches to promote the “Southward Planting of Herbaceous Peony” and achieve the “renaissance” of herbaceous peony in the Zhejiang landscape^7,30^. The resource introduction and performance evaluation of the northern and southern peonies are both imperative studies^3,6^.

From 2012 to 2017, we introduced 29 cultivars from northern China and the special southern ‘Hang Baishao’ to Hangzhou, the provincial capital of Zhejiang Province, and then carried out systematic research, such as quantitative observations of annual growth cycles, experiments to test heat resistance under natural and artificial high temperatures, crossbreeding between these northern and southern cultivars and measurements of their hybrid progenies. In this study, we reported all these six-year data collected from long-running quantitative observation (LQO) and combined them into an evaluation system constructed by one of the MCDM approaches, i.e., the AHP. Then, we used the “LQO + AHP” strategy to evaluate the comprehensive performance of 30 herbaceous peony cultivars under the hot-humid climate^12,31–33^. These studies could provide the abundant practical experience necessary to promote the “Southward Planting of Herbaceous Peony”, and could also show the dependability, objectivity and comprehensiveness of the strategy integrating the MCDM approach and the accurate data from LQO in the evaluation of ornamental and other economic plants after being introduced to other areas beyond their original habitats^12,14,34^.

## Results

### LQO of annual growth cycles for six years: bud sprouting and growth

**Figure 1:**
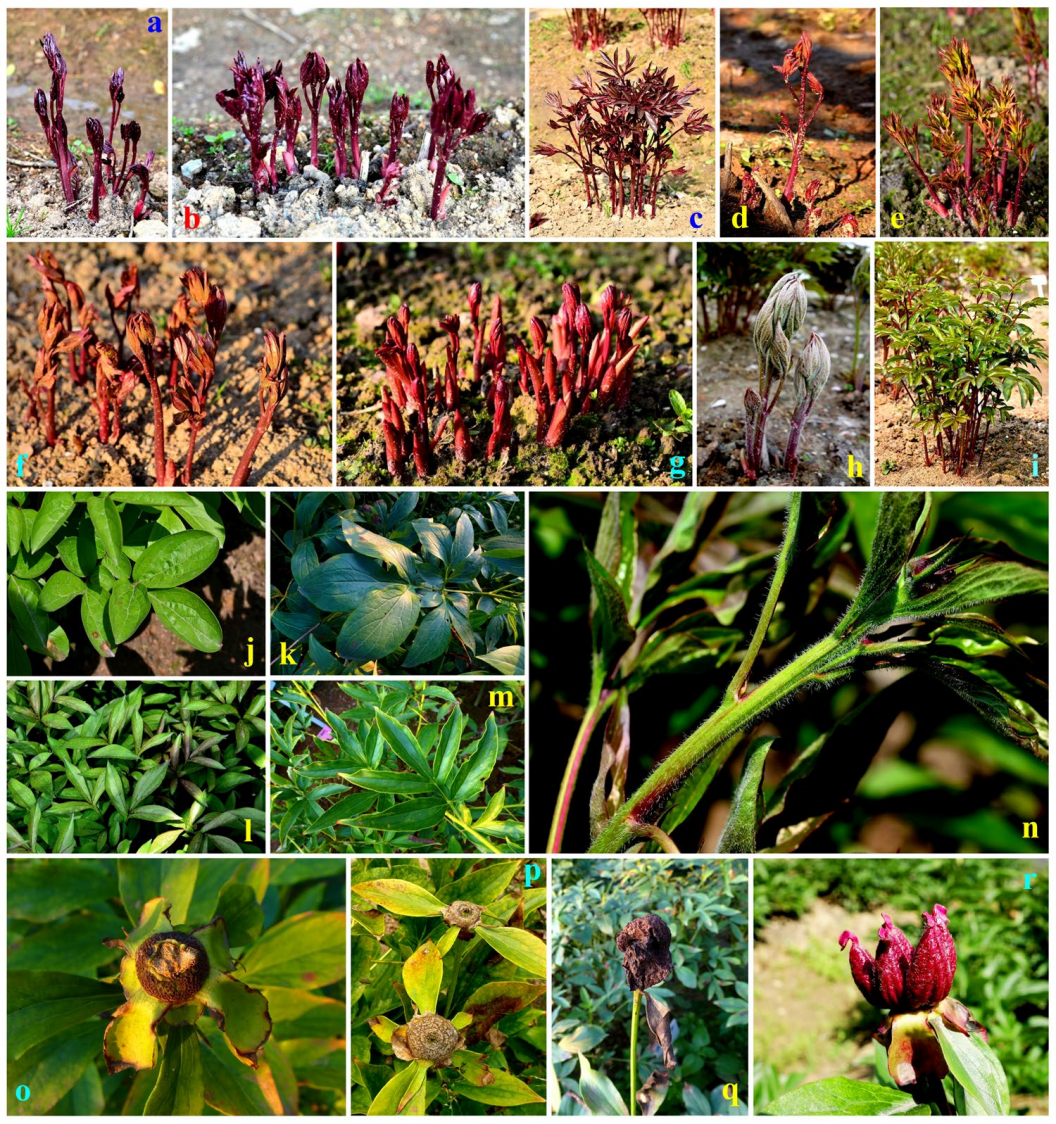
Bud sprouting, stem and leaf growth and fruiting of partial cultivars. (**a**–**c**) purple red TBSL of ‘Xuanli Duocai’, ‘Qiaoling’ and ‘Die Lianhua’, respectively; (**d**–**f**) orange red or yellow green TBSL of ‘Zhuguang’, ‘Qingwen’ and ‘Qingyun Hong’, respectively; (**g**) rosy red TBSL of ‘Guanyin Zuo’; (**h**) special TBSL of ‘Yangfei Chuyu’ covered by thick white hairs; (**i**) purple red stems and yellow green new leaves of ‘Zifeng Chaoyang’ (cultivars in (**a**–**i**) were all acquired high points in the ornamental duration of TBSL in Supplementary Table S16); (**j**,**k**) broadly ovate leaves of ‘Da Fugui’ and ‘Lanju’ with a low ratio of length to width (Supplementary Table S7); (**l**,**m**) narrow leaves of ‘Guanyin Zuo’ and ‘Meiju’ with a high ratio of length to width (Supplementary Table S7); (**n**) mature leaves and stems of ‘Yangfei Chuyu’ still covered by thick white hairs; (**o**,**p**) ‘Die Lianhua’ never produced any fruits, and immature fruits of ‘Linghua Chenyu’ dropped early (Supplementary Table S15), so they left only the disk-shaped fruit receptacles; (**q**) withered flowers of ‘Qingwen’ were not dropped and always wrapped receptacles and flowering stems. ‘Die Lianhua’, ‘Linghua Chenyu’ and ‘Qingwen’ were all scored as having a low ornamental value of ripe fruit in Supplementary Table S16); (**r**) fascinating vivid red fruits of ‘Qihua Lushuang’, which acquired the same high point as that of ‘Hang Baishao’ in terms of the ornamental value of ripe fruit (Supplementary Table S16).

**Figure 2:**
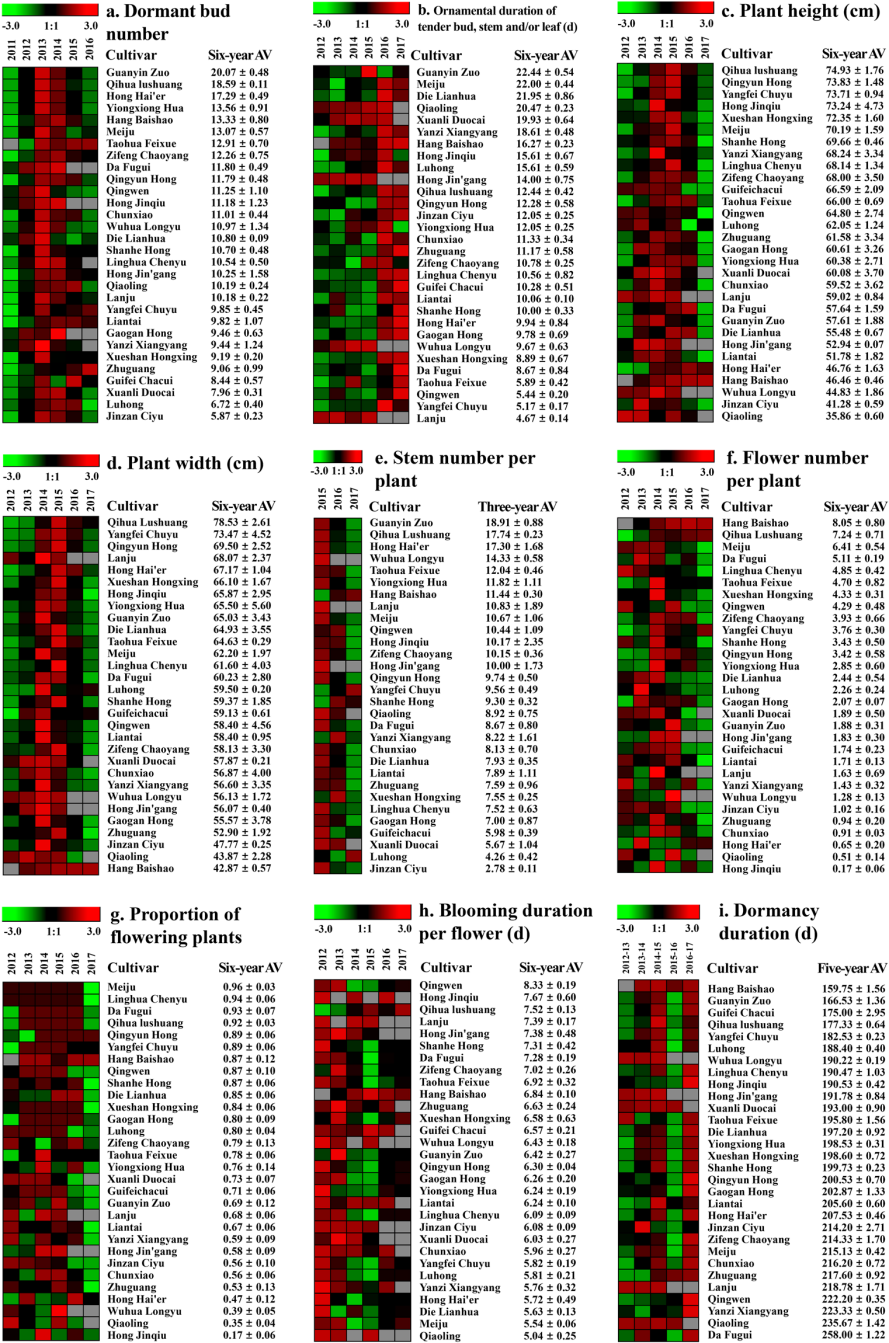
The heatmap group illustrating the change trend of nine important morphologies over six experimental years from 2011–2012 to 2016–2017. Bars on the top of the nine heatmaps indicate the change range of phenotypic data. Red cells mean increase and green cells mean decrease; gray cells mean uncollected or nonexistent data because all plants died, no flowers appeared, or one cultivar had not yet been introduced in observation time (‘Hang Baishao’, 2012). Notes of “−3.0 1:1 + 3.0” under bars indicate the phenotypic values normalized by the software named Genesis 1.7.6. The first column on the right side of each heatmap shows the corresponding cultivar names, and the second column shows the six-year average values (AV) of one phenotypic index. The Supplementary Tables in the Supplementary Data corresponding to the nine heatmaps of **a**, **b**, **c**, **d**, **e**, **f**, **g**, **h** and **i** are S2, S3, S4, S5, S6, S8, S9, S13 and S19 respectively.

The basic information of 30 cultivars is listed in Supplementary Table S1 of the Supplementary Dataset (an Excel file including two sheets, five sections and 37 Supplementary Tables). Dormancy period was regarded as an initiation of annual growth cycle before bud sprouting (Fig. 1a–h). Therefore, the number of dormant buds were recorded in six autumns and firstly showed in Supplementary Table S2. Most cultivars increased buds in the first four years while declined in the last two years (Fig. 2a and Supplementary Table S2).

Then, the initial period of more than 50% bud sprouting (IBS) was adopted instead of the initial period of the first bud sprouting, which could reflect the global sprouting performance (Supplementary Table S3). The southern ‘Hang Baishao’ and northern ‘Hong Jinqiu’ were the two cultivars that sprouted the earliest. The tender bud, stem and/or leaf (TBSL) of different cultivars were purple red, orange red, yellow green, rosy red or even covered by thick white hairs, which had special ornamental value (Fig. 1a–i). ‘Guanyin Zuo’, ‘Meiju’, ‘Die Lianhua’ and ‘Qiaoling’ had the longest ornamental durations of the TBSL, with more than 20.00 days, and the TBSL-ornamental durations of most cultivars increased significantly in 2016 and 2017 according to Fig. 2b and Supplementary Table S3. This trait will be one of three sub-indices used to evaluate the index *C*_7_ in the subsequent analysis (Supplementary Table S16).

Observations of plant height and width, stem number and leaf shape can provide information on the peony application in parks and gardens. ‘Qihua Lushuang’, ‘Yangfei Chuyu’ and ‘Qingyun Hong’ had high plant heights and widths, while ‘Qiaoling’ was the shortest cultivar with very limited plant width (Supplementary Tables S4 and S5). The plant heights and widths of most cultivars increased obviously from 2013 to 2015 and then decreased in the last two years (Fig. 2c,d). ‘Guanyin Zuo’ was a cultivar with a typical tufty appearance because of the maximum ratio of stem number to diameter (Supplementary Table S6). The stem numbers of most cultivars decreased obviously in the last two years (Fig. 2e). Additionally, different cultivars had different leaf shapes, lengths and widths (Fig. 1j–n, Supplementary Table S7), which showed completely different appearances in all plants.

### LQO: flowering performances

**Figure 3:**
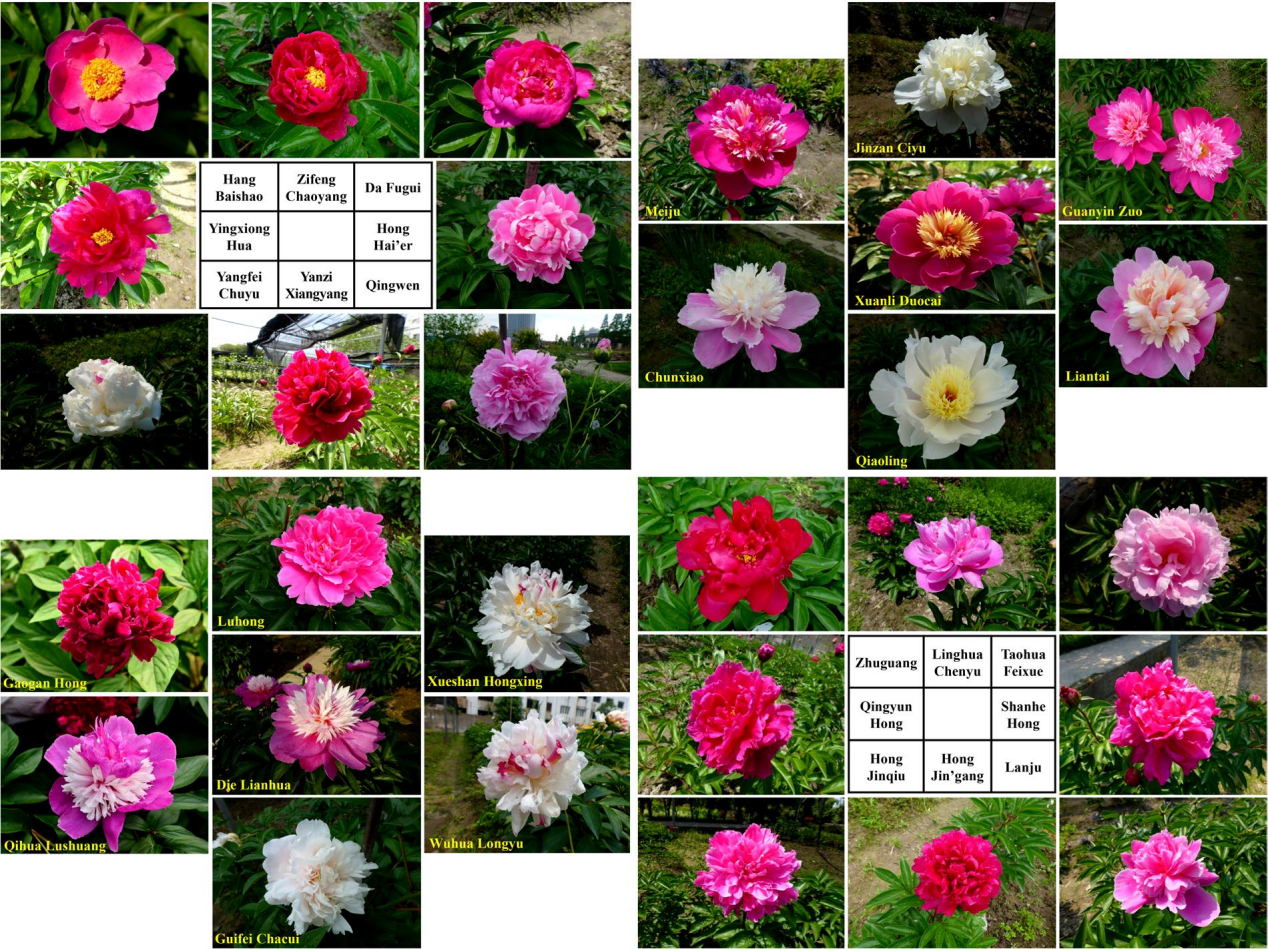
Flowers at the full blooming stage of 30 *P. lactiflora* cultivars introduced from the south and north of China.

The flower types and colors of 30 herbaceous peony cultivars are shown in Fig. 3 and Supplementary Table S1. ‘Hang Baishao’, ‘Qihua Lushuang’ and ‘Meiju’ performed best in several important flowering indices, such as flower number per plant (FNP), proportion of flowering plant (PFP) and value of opened flower number minus aborted flower number per plant (OMA), while ‘Hong Jinqiu’, ‘Qiaoling’ and ‘Hong Hai’er’ performed very bad in these crucial indices (Supplementary Tables S8, S9 and S11). ‘Hang Baishao’ also had the second lowest number of aborted flowers, which means most of the ‘Hang Baishao’ flower buds could bloom and were not disturbed by the rapid-rising temperature in Hangzhou spring (Supplementary Table S10). The FNP and PFP of most cultivars peaked in 2014 and 2015 (Fig. 2f,g).

‘Guanyin Zuo’, ‘Qiaoling’ and ‘Hang Baishao’ had strong upright flowering stems (Supplementary Table S12). The initial periods of more than 30% flower blooming (IFB) of ‘Hang Baishao’, ‘Hong Jinqiu’ and ‘Xuanli Duocai’ were earlier than those of the other 27 cultivars, which bloomed before April 20 (Supplementary Table S13). ‘Qingwen’ was the only cultivar that bloomed longer than 8.00 days per flower. Additionally, the flowering durations shortened each year, except for ‘Qihua Lushuang’ and ‘Hang Baishao’ (Fig. 2h). Additionally, Supplementary Table S14 shows the information relevant to stamen and pistil, which was used to determine the appropriate role of each cultivar in crossbreeding (Supplementary Table S22).

### LQO: fruiting and withering

The fruits of most cultivars could not be appreciated, except for ‘Qihua Lushuang’ and ‘Hang Baishao’ because of their beautiful red ripe fruits (Supplementary Table S15, Fig. 1o–r). The initial periods of more than 50% leaves losing green/withering in autumn (ILL) reflected the persistence of green leaves (Supplementary Table S15). As shown in Supplementary Table S16, the ornamental duration of the TBSL, ornamental value of ripe fruit, and green-leaf duration were adopted as three sub-indices used to evaluate the index *C*_7_ named “other ornamental values”.

### Heat resistance determination: under natural high temperatures

**Figure 4:**
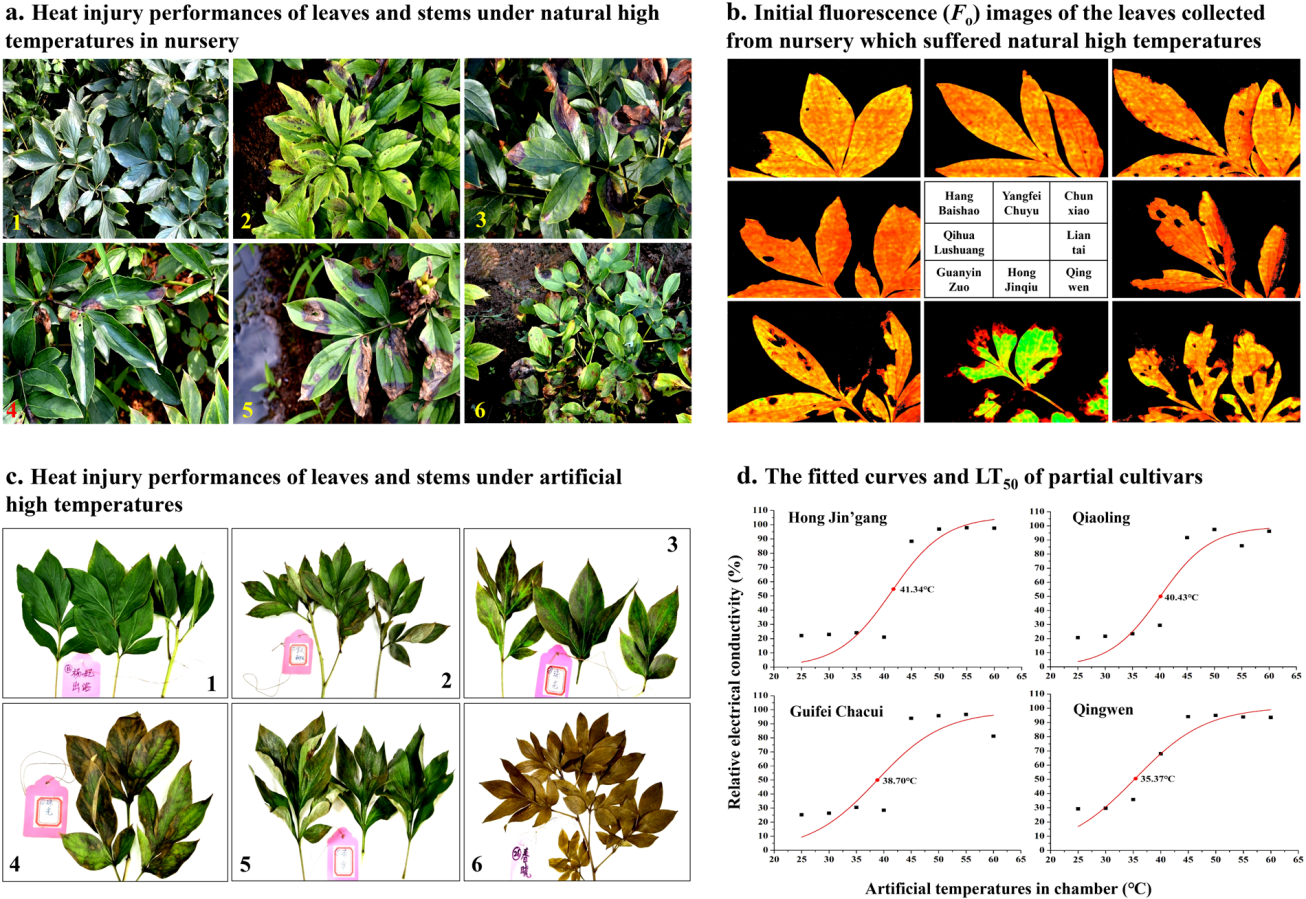
Determination of heat resistance of 30 herbaceous peony cultivars under natural and artificial high temperatures.

**Figure 5:**
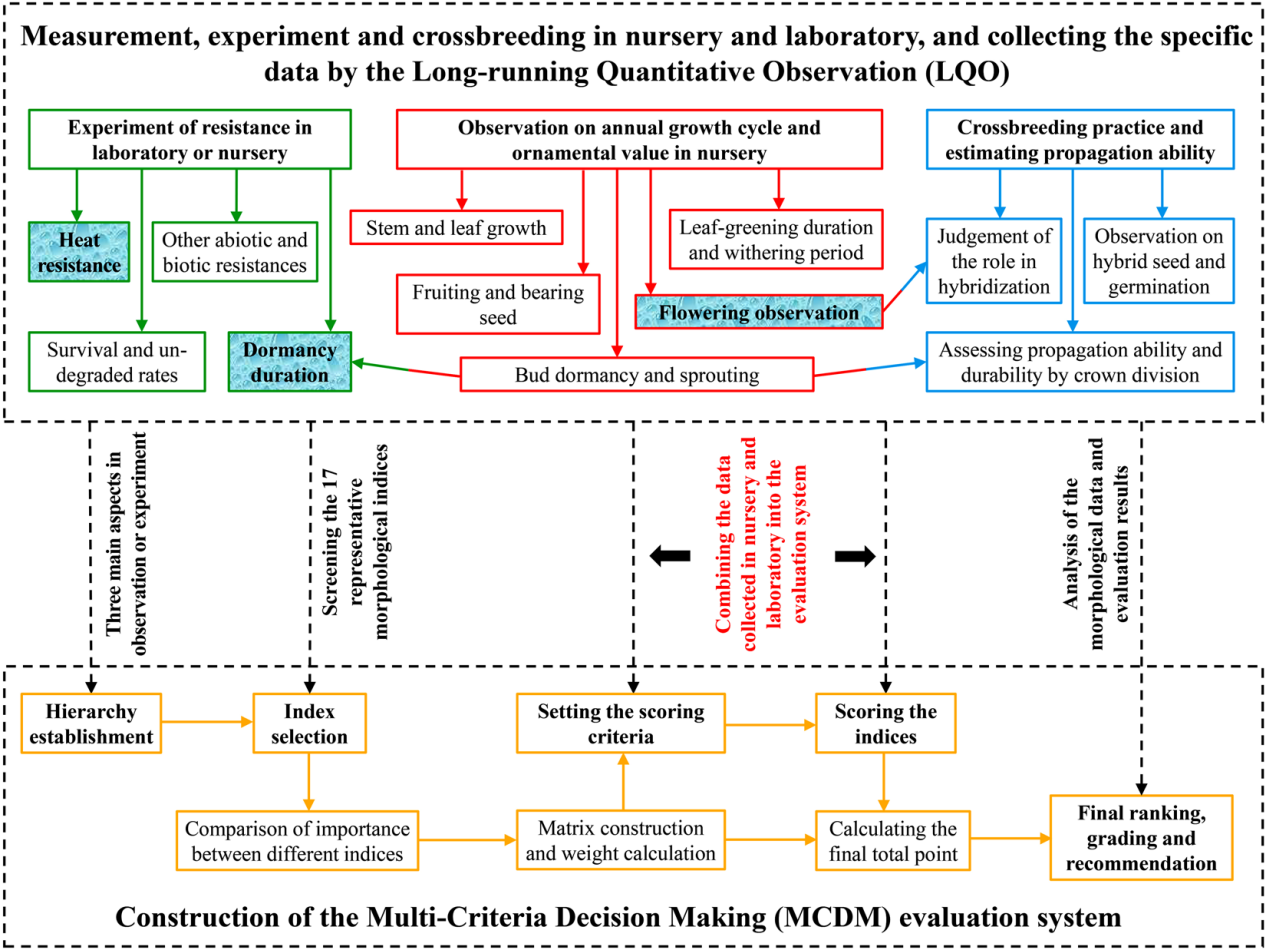
Evaluation strategy integrating the “LQO + MCDM”, and related indispensable observations/experiments for evaluating the comprehensive performance of the target economic plants to be cultivated beyond “the most comfortable region”.

High temperatures in July and August of 2013 year broke the historical record for Hangzhou during the last 50 years (Supplementary Table S37 in the sheet 2 of Supplementary Dataset)^35–37^. Only a few cultivars performed well under the scorching heat in August, such as ‘Hang Baishao’ and ‘Yangfei Chuyu’, which had relatively low values of the heat injury index (HII) (Table 1, Supplementary Table S17, Fig. 4a-1). However, most of the cultivars suffered severe heat injuries that mainly manifested as yellow-green leaves with small and dense sunburned perforations (Fig. 4a-2), large black-brown patches on leaves (Fig. 4a-3,a-4), fragmented and parched leaves (Fig. 4a-5), and withered or died stems and whole plants (Fig. 4a-6). Figure 4b and Supplementary Table S18 show the initial fluorescence (*F*_o_) images and chlorophyll fluorescence parameters for eight cultivars, which were consistent with the HII and RCC data and performance of leaves and stems in the nursery.

**Table 1:** Determination of resistances to heat, disease and pests, and proportions of survival and non-degraded plants after six-year cultivation period.

Cultivars	Heat injury indices (HII)	Relative chlorophyll contents (RCC)	Semilethal temperatures (LT_50_)	Levels of disease and pest resistances	Proportions of survival plants	Proportions of non-degraded plants
Chunxiao	0.28 ± 0.02	52.80 ± 0.82	39.78 ± 0.04	4.57 ± 0.06	0.89 ± 0.19	0.56 ± 0.51
Da Fugui	0.61 ± 0.06	19.18 ± 4.01	40.38 ± 0.41	1.35 ± 0.14	0.78 ± 0.19	0.55 ± 0.39
Die Lianhua	0.32 ± 0.07	24.59 ± 3.46	40.80 ± 0.21	3.28 ± 0.31	1.00 ± 0.00	0.78 ± 0.39
Gaogan Hong	0.28 ± 0.07	32.40 ± 1.71	40.84 ± 0.63	3.60 ± 0.34	0.78 ± 0.19	0.45 ± 0.39
Guanyin Zuo	0.40 ± 0.13	44.51 ± 1.41	40.16 ± 0.82	4.62 ± 0.21	0.67 ± 0.00	0.56 ± 0.20
Guifei Chacui	0.39 ± 0.06	46.97 ± 5.04	39.55 ± 0.85	3.96 ± 0.09	0.78 ± 0.19	0.78 ± 0.19
Hang Baishao	0.18 ± 0.04	58.65 ± 0.85	41.68 ± 0.16	4.76 ± 0.14	1.00 ± 0.00	1.00 ± 0.00
Hong Hai’er	0.37 ± 0.09	33.51 ± 4.63	41.50 ± 0.36	3.35 ± 0.47	0.78 ± 0.19	0.78 ± 0.19
Hong Jin’gang	0.25 ± 0.04	52.86 ± 2.70	41.27 ± 0.34	4.94 ± 0.10	0.00 ± 0.00	0.00 ± 0.00
Hong Jinqiu	0.37 ± 0.26	30.63 ± 4.18	40.26 ± 0.23	3.10 ± 0.25	0.56 ± 0.20	0.33 ± 0.34
Jinzan Ciyu	0.36 ± 0.08	34.73 ± 2.09	41.35 ± 0.22	3.19 ± 0.09	1.00 ± 0.00	0.00 ± 0.00
Lanju	0.46 ± 0.07	50.88 ± 1.80	40.41 ± 0.23	4.89 ± 0.05	0.00 ± 0.00	0.00 ± 0.00
Liantai	0.34 ± 0.14	43.90 ± 1.67	41.26 ± 0.40	3.57 ± 0.10	0.78 ± 0.19	0.56 ± 0.20
Linghua Chenyu	0.46 ± 0.07	28.55 ± 1.94	40.97 ± 0.54	3.81 ± 0.03	1.00 ± 0.00	0.67 ± 0.34
Luhong	0.37 ± 0.09	48.23 ± 1.89	40.50 ± 0.16	4.53 ± 0.07	0.44 ± 0.20	0.44 ± 0.20
Meiju	0.41 ± 0.12	46.52 ± 4.03	40.71 ± 0.20	3.78 ± 0.06	1.00 ± 0.00	0.78 ± 0.19
Qiaoling	0.42 ± 0.18	18.17 ± 2.62	39.85 ± 0.24	1.84 ± 0.25	0.00 ± 0.00	0.00 ± 0.00
Qihua Lushuang	0.41 ± 0.06	46.91 ± 1.40	41.00 ± 0.33	4.90 ± 0.06	1.00 ± 0.00	0.78 ± 0.19
Qingwen	0.57 ± 0.14	33.35 ± 4.20	35.10 ± 0.46	1.56 ± 0.15	0.78 ± 0.19	0.56 ± 0.51
Qingyun Hong	0.46 ± 0.01	21.35 ± 3.54	41.20 ± 0.28	4.03 ± 0.21	0.78 ± 0.19	0.78 ± 0.19
Shehe Hong	0.29 ± 0.16	31.81 ± 3.64	41.47 ± 0.15	3.58 ± 0.09	1.00 ± 0.00	0.78 ± 0.19
Taohua Feixue	0.31 ± 0.10	32.57 ± 1.27	40.19 ± 0.06	3.79 ± 0.34	1.00 ± 0.00	1.00 ± 0.00
Wuhua Longyu	0.44 ± 0.08	30.11 ± 1.68	40.23 ± 0.17	2.97 ± 0.13	0.00 ± 0.00	0.00 ± 0.00
Xuanli Duocai	0.46 ± 0.07	35.43 ± 4.86	40.69 ± 0.52	4.42 ± 0.43	0.00 ± 0.00	0.00 ± 0.00
Xueshan Hongxing	0.48 ± 0.10	36.53 ± 2.51	39.74 ± 0.40	3.05 ± 0.19	0.78 ± 0.19	0.56 ± 0.20
Yangfei Chuyu	0.16 ± 0.10	50.70 ± 2.11	41.12 ± 0.59	4.83 ± 0.06	1.00 ± 0.00	0.89 ± 0.19
Yanzi Xiangyang	0.32 ± 0.10	31.50 ± 1.85	41.21 ± 0.08	3.50 ± 0.19	1.00 ± 0.00	0.78 ± 0.39
Yiongxiong Hua	0.34 ± 0.07	31.56 ± 3.00	40.43 ± 0.37	3.41 ± 0.28	1.00 ± 0.00	0.55 ± 0.39
Zhuguang	0.48 ± 0.10	21.59 ± 2.60	40.69 ± 0.56	2.60 ± 0.10	0.67 ± 0.34	0.00 ± 0.00
Zifeng Chaoyang	0.42 ± 0.04	31.80 ± 5.60	40.86 ± 0.51	3.18 ± 0.32	1.00 ± 0.00	0.55 ± 0.39

### Heat resistance determination: under artificial high temperatures

Leaves were still green and uninjured after being exposed to 25, 30 and 35 °C (Figs. 4c-1), and then they became injured at temperatures higher than 40 °C. Because the constant temperature and humidity chambers could only provide heat but not sunburn, the injured performances caused by artificial high temperatures were significantly different from those caused by natural high temperatures, which mainly included four types: (1) no sunburned perforations; (2) injured parts emerged evenly on leaf surface; (3) leaf surface was very damp and slimy because of tissue fluid extravasation; and (4) completely withered but intact leaves without any fragmented parts even at a temperature of 60 °C (Fig. 4c-2 to c-6). The fitted curves of the relative electrolytic leakage values for partial cultivars are shown in Fig. 4d. Temperatures on the y-axis corresponding to the inflection point of the “S” curves were the LT_50_ values. According to Table 1 and Supplementary Table S17, the LT_50_ of ‘Hang Baishao’ was 41.68 °C, while the LT_50_ of ‘Qingwen’ was 35.10 °C; these values represented the highest and lowest values among the 30 cultivars, respectively.

Based on all experiments using the natural and artificial heat treatments, three sub-indices, i.e., HII, RCC and LT_50_, were adopted to evaluate the comprehensive heat resistances of 30 herbaceous peony cultivars (Supplementary Table S17). ‘Hang Baishao’, ‘Hong Jin’gang’ and ‘Yangfei Chuyu’ were the three most heat-resistant cultivars, while ‘Qingwen’, ‘Da Fugui’ and ‘Qiaoling’ were the three most heat-sensitive cultivars.

### Determination of dormancy duration, disease and pest resistance, survival and non-degraded rates after six years of cultivation

‘Hang Baishao’ needed only 159.75 days of dormancy, making it the only cultivar with a dormancy period shorter than 160.00 days (Supplementary Table S19, Fig. 2i). ‘Da Fugui’ required 258.00 days to finish the entire dormancy period, which was longer than that of ‘Hang Baishao’ by approximately 100.00 days. These data showed that ‘Hang Baishao’ probably had a low chilling requirement (CR) to break bud dormancy. According to Supplementary Table S20, ‘Hong Jin’gang’, ‘Qihua Lushuang’ and ‘Lanju’ had few diseases and pests. After six years of cultivation, ‘Die Lianhua’, ‘Hang Baishao’ and the other 10 cultivars had 100% survival rates, while plants of ‘Hong Jin’gang’, ‘Lanju’ and the other three cultivars all disappeared. Only two cultivars, i.e., ‘Hang Baishao’ and ‘Taohua Feixue’, did not show obvious signs of degradation (Supplementary Table S21).

### Hybridization between the southern and northern cultivars

The number of hybrid seeds per pollinated flower (NSP), proportion of seed-bearing flowers after hybridization (PSF) and germination rate of hybrid seeds (GRS) were adopted to evaluate the ability to participate in crossbreeding for one cultivar as a female parent (Supplementary Table S22). Combinations of ‘Hang Baishao’ × ‘Meiju’, ‘Hang Baishao’ × ‘Shanhe Hong’ and ‘Hang Baishao’ × ‘Qihua Lushuang’ had the maximum NSP values, producing more than 10.00 hybrid seeds in each pollinated flower (Supplementary Table S22).

### Evaluation of the comprehensive performance of 30 cultivars by the strategy integrating the LQO and MCDM approach: selection of indices and construction of AHP system

A comprehensive AHP evaluation system was constructed after hierarchy design, index selection, judgement matrix construction, consistent examination and evaluation weight assignment (Supplementary Tables S23–S32). The final completed system is shown in Table 2 and Supplementary Table S33. “Ornamental values (*B*_1_)”, “Resistances and dormancy duration (*B*_2_)” and “Abilities to propagate and create new germplasm (*B*_3_)” were selected as the three indices in the constrained layer. “Ornamental values (*B*_1_)” was regarded as the most important index with the highest weight (0.5396). “Flower number per plant” (*C*_1_) and “Proportion of flowering plant (*C*_2_)” had the highest weights (0.1372) among the 17 layer indices of *C* to *A*.

**Table 2:** Construction of the evaluation system and different index weights.

Target layer	Weights	Constrained layer	Weights	Index layer	Weights of layer *C* to *A*
Evaluation for the comprehensive performance of herbaceous peonies in Hangzhou (*A*)	1.0000	Ornamental values (*B*_1_)	0.5396	Flower number per plant (*C*_1_)	0.1372
				Proportion of flowering plant (*C*_2_)	0.1372
				Upright level of flowering stem (*C*_3_)	0.0801
				Flowering duration per flower (*C*_4_)	0.0801
				Flowering period (C_5_)	0.0474
				Fragrance level (C_6_)	0.0288
				Other ornamental values (C_7_)	0.0288
		Resistances and dormancy duration (*B*_2_)	0.2969	Heat Resistance (*C*_8_)	0.0884
				Dormancy duration (*C*_9_)	0.0884
				Disease and pest resistances (*C*_10_)	0.0264
				Final proportion of survival plant after six-year cultivation (*C*_11_)	0.0468
				Final proportion of non-degraded plant after six-year cultivation (*C*_12_)	0.0468
		Abilities to propagate and create new germplasm (*B*_3_)	0.1634	Propagation ability by crown division (*C*_13_)	0.0467
				Durability of propagation ability by crown division (*C*_14_)	0.0467
				Potential to be male parent in hybridization (*C*_15_)	0.0233
				Potential to be female parent in hybridization (*C*_16_)	0.0233
				Ability to produce seed in pollinated flower (*C*_17_)	0.0233

### Final ranking, grading and recommendations

Details in scoring and ranking the 17 indices are presented in Supplementary Tables S34 and S35, and the final evaluation results are shown in Table 3 and Supplementary Table S36. ‘Qihua Lushuang’ acquired the highest point values of 92.88, and ‘Hang Baishao’ was ranked second, with 91.94 points, which are the only two cultivars with more than 90.00 points. The other 28 cultivars all had fewer than 80.00 points. At the bottom of the ranking list, ‘Qiaoling’ scored the lowest, with a point value of 35.09. Additionally, the ranking list of 30 cultivars was divided into five grades (I to V, Supplementary Table S36 and Table 3), which represented the different recommendation levels regarding the landscape application of herbaceous peony in the relative low-latitude regions.

**Table 3:** Final evaluation results of the comprehensive performance of 30 peony cultivars planting in the central subtropical city Hangzhou.

Cultivars	Final total point values	Final ranking	Final grading
Qihua Lushuang	92.88	1	I
Hang Baishao	91.94	2	I
Yangfei Chuyu	79.84	3	I
Meiju	78.58	4	I
Shanhe Hong	77.53	5	I
Taohua Feixue	74.98	6	I
Linghua Chenyu	70.34	7	II
Guanyin Zuo	68.65	8	II
Qingyun Hong	68.36	9	II
Zifeng Chaoyang	63.89	10	II
Yiongxiong Hua	63.13	11	II
Luhong	62.97	12	II
Da Fugui	62.93	13	III
Die Lianhua	62.42	14	III
Gaogan Hong	60.47	15	III
Xueshan Hongxing	60.19	16	III
Liantai	58.27	17	III
Guifei Chacui	58.12	18	III
Qingwen	57.37	19	IV
Hong Jin’gang	56.68	20	IV
Xuanli Duocai	53.77	21	IV
Yanzi Xiangyang	53.12	22	IV
Chunxiao	51.84	23	IV
Jinzan Ciyu	50.46	24	IV
Hong Jinqiu	47.94	25	V
Lanju	47.25	26	V
Hong Hai’er	44.78	27	V
Wuhua Longyu	41.04	28	V
Zhuguang	36.84	29	V
Qiaoling	35.09	30	V

## Discussion

Each plant species is locally adapted and has “the most comfortable region” for its planting and growth, where it has the same or similar climate and ecological environment as that found in its natural distribution area in most cases^38^. Plants can perform best in terms of flowering and fruiting and can create the highest economic value in this ideal region^4^. However, most species can actually be planted in much wider areas beyond “the most comfortable region” because of their potential adaptability and lifelong plasticity, which must be exploited by artificial introduction and resistance training^2–4,6,39^. The planting area is often enlarged across altitudes or latitudes, such as the “downward planting of alpine primroses, gentians or rhododendrons”^6,18^, “northward planting of Mei Flower”^8,40,41^ and “southward planting of herbaceous and tree peonies”^7,11,22–28^ in the Northern Hemisphere. The global warming trend facilitates the success of “northward planting” but introduces many obstacles to promoting target plants with winter dormancy traits from high to low elevations or northern to southern areas, such as herbaceous peony^1,10,42^. Although some advanced technologies could help investigate the potential suitable areas for herbaceous or tree peony cultivation spanning an entire country^2^, many years of hard work on resource introduction, resistance determination, crossbreeding and comprehensive evaluation are indispensable for truly realizing the “Southward Planting of Herbaceous Peony”^3,6^.

The screening of the superior cultivars and the selection of the appropriate parents to perform crossbreeding are two main approaches to enlarge the planting area of the target plant, and both approaches should be based on the resource evaluation^4,6^. In this study, we adopted the AHP to evaluate the peony performances, which had been confirmed as a classic and highly effective MCDM methodology to evaluate the specific goals^14,32,33^. This method has been widely used in many scientific fields, such as astronomy, geography, energy science, environmental ecology, healthcare, management and engineering^13,34,43–48^. Furthermore, the AHP has been applied to evaluate the important traits or comprehensive performances of economic plants in the fields of agronomy, forestry, biology and ornamental horticulture^4–6,12,14,15,49–53^.

When the AHP system is constructed, index selection and scoring criterion should be objective and based on the actual requirement and specific data rather than on subjective judgment^4,49,51^. For example, in this study, most of the indices could be quantified, and most of the evaluation results were supported by accurate data, which were collected either from serious LQO or from multiple experiments (Fig. 5)^6,12,14^. To ensure the objectivity, data of each index were ranked and scored by numerical values (such as *C*_1_ in Supplementary Table S8) or phenotypic traits (*C*_3_ and *C*_6_ in Supplementary Table S12), and different cultivars with the same values or traits were assigned the same ranks and scores (*C*_11_ in Supplementary Table S21). Additionally, partial indices were compound including several sub-indices, such as *C*_7_ and *C*_8_ (Supplementary Tables S16 and S17), which covered as many factors as possible and avoided the one-sidedness and subjectivity only from one aspect. However, it is also difficult to thoroughly avoid subjectivity, such as “Fragrance level (*C*_6_)” (Supplementary Table S12), which was hard to quantify (Supplementary Table S33)^6^. The subjectivity was difficult to eliminate completely in pair-wise comparisons of the relative importance in the construction of judgement matrices (Supplementary Tables S24–S29). Therefore, in our opinion, objectivity is relative but not absolute in evaluation studies, regardless of which approach or system is adopted^14,34^.

The construction of a completed MCDM evaluation system includes some common steps, such as choosing indices, making criteria and assigning weights^13,14,32^ (Fig. 5). First, the completion of these steps should be guided by the specific purpose, characteristics of the target plant and expert suggestion^4,12,14,15^. In this study, the index of “Ornamental values (*B*_1_)” was assigned the highest weight in the constrained layer, and most of the indices under *B*_1_ were directly related to the ornamental value of the flower (Supplementary Tables S23, S33 and S34). These results are reasonable because the purpose of this study was to transport the peony to a more southern area as a landscape plant but not as a medicinal, edible or oil plant^54,55^. The flower is the most important ornamental organ of the peony and should definitely receive considerable attention^41^ (Fig. 3). Additionally, the TBSL of both herbaceous and tree peonies are fascinating ornamental organs because of anthocyanin biosynthesis, and thus they can provide significant landscape benefits to the early-spring park and garden^56^. Therefore, the ornamental value of the TBSL was also involved in the three sub-indices of “Other ornamental values (*C*_7_)” (Supplementary Tables S33 and S34, Fig. 1), to prevent the omission of secondary ornamental organs and thus ensure the comprehensiveness of the evaluation, although the weight of this index was not sufficiently high (0.0288).

The data not included in the evaluation system are also useful and deserve attention. For example, the aborted flower number and OMA (Supplementary Tables S10 and S11) were two valuable morphologies but were not included in the evaluation system. The abortion of floral organs is a frequent and troublesome problem in peony cultivation and greatly inhibits flower production^20,57,58^. The number of aborted flowers would decrease, while healthy flowers would increase correspondingly if the environment was appropriate and the nutrient transformation was normal. Therefore, the aborted flower number and OMA could reveal the flowering potential of different cultivars that were planted in low latitudes^20,57–59^, although these data were not adopted into the AHP system.

Additionally, the change tendency of nine important indices over six years were also not used directly in evaluation, but could reflect the dynamic trends in terms of sprouting, growth, flowering and dormancy (Fig. 2). Partial indices presented parabolic trends during six years (Fig. 2a,c,d,f), their values decreased significantly during the fifth to sixth years after introducing to Hangzhou (2016 to 2017, eight- to nine-year-old peony crowns). The main reasons are the muggy climates in springs, summers and winters of Hangzhou, which were further analyzed in the following content.

Second, the establishment of the completed evaluation system should consider the local environment and climate of the target *ex situ* planting area^4^. For herbaceous peony, high temperatures in summers and winters are two main obstacles for planting in low latitudes^29,30,60^. Rapid warming during spring leads to the severe lodging of the inflorescence stem because of the excessively rapid growth and weak mechanism strength^61–64^. Then, the scorching summer causes serious heat damage to stems and leaves, which reduces the photosynthetic efficiency and leads to growth inhibition and plant dehydration^11,29,30,64^. Therefore, the two indices of “Upright level of flowering stem (*C*_3_)” and “Heat Resistance (*C*_8_)” were chosen to evaluate the peony performance under high temperatures in spring and summer^60,62,63^. Two experiments with natural and artificial heat treatments and relevant measurements in the hottest summer of 2013 reflected the maximum heat injury and tolerance of 30 peony cultivars in the central city of Hangzhou (Fig. 4, Supplementary Table S17)^60,65–68^.

Compared with the hot summer, the warm winter causes more profound and multiple negative influences to herbaceous peony^3,10^. Warming winters hinder the fulfillment of the chilling requirement (CR), which is a decisive trait for both herbaceous and tree peonies that are to be successfully planted in low latitudes^1,24,58,65,66^. The lack of chilling accumulation during the last winter negatively affects all subsequent growth and development in the following spring, such as an incomplete overwintering bud dormancy release, uneven sprouting, abnormal growth and poor blooming^3,25,57,66,67^. Finally, underground crowns degrade within two to four years, and then overwintering buds wither and the whole plants die soon after introduction^7,18^. Thus, adequate chilling accumulation, sufficient dormancy duration and regular seasonal growth cycles are crucial for herbaceous peony to adapt to warm winters and ensure ornamental and other economic values^18,68,69^. Therefore, “Dormancy duration (*C*_9_)” was certainly adopted and acquired the same high weight (0.0884) as that of “Heat Resistance (*C*_8_)”, which could reflect the potential CR trait of peony to some extent^1,58,70^. A short dormancy duration and low CR are certainly great traits for herbaceous peony that is to be planted in lower latitudes^3^. However, a short dormancy duration cannot completely represent the low CR, and a long dormancy duration is not symbolic of a high CR trait. Sensitivity to photoperiod and cold, leaf senescence and winter hardiness also determine the dormancy duration, foliar green and bud sprouting periods^3,19,21,71–73^. The exact value of CR should be measured by chilling treatments and regrowth experiments^70,74,75^, which have already been reported in our previous publication^7^. Then, screening the cultivars with the significantly low CR trait is crucial work for achieving the “Southward Planting of Herbaceous Peony”^3^.

Additionally, waterlogging induced by rainy and humid climate, and the pH values in soil and irrigation water are also restriction factors^17^. However, it is impossible to cover all potential stresses in one evaluation system; thus, we designed two “ultimate” indices (*C*_11_ and *C*_12_) to show the final performance of 30 cultivars after suffering various abiotic and biotic stresses without any plant or crown replacing after cultivation for six continuous years.

Third, there are another two factors that have often been neglected in previous studies evaluating economic plants, those are, the “abilities to propagate and create new germplasm” of the target plant in *ex situ* planting area^51^. These two factor are closely related to sustainable development and germplasm innovation for cultivating economic plant in the area beyond “the most comfortable region”, which is the motivation of setting the index *B*_3_ (Supplementary Tables S23, S33 and S34) in this study^12^. Ornamental peonies are mainly propagated by crown division instead of sowing seed. The segregation of characters will occur in the offspring by seed propagation. In the most cases, one mature mother crown with more renewal buds could be divided to more offspring crowns, each of which has one bud at least, and can bloom rapidly in the next spring. Therefore, to some extent, bud number of peony can reflect the ability to propagate by crown division. In this study, we designed two indices (*C*_13_ and *C*_14_) to evaluate the ability and durability of division-propagation based on the six-year bud observation (Supplementary Tables S2). The relevant results could provide the reference to the large-scale production and sustainable application of the excellent peonies in Hangzhou.

Additionally, we observed the pistils and stamens, and then performed the crossbreeding practice between the southern and northern cultivars (Fig. 3, Supplementary Tables S14 and S22). These data were used to evaluate the indices *C*_15_, *C*_16_ and *C*_17_, which could contribute to the reasonable selection of parent-pairs and a better understanding of the potential for creating new germplasm from different combinations^12^. The completed crossbreeding study should include subsequent observations on the mature F1 and F2 progenies, and also involve the study on the progenies acquired in backcrosses between progenies and original parents, which is a topic that requires further research^12,51,76^.

As for the evaluation results, the top of the final list was not the unique southern peony ‘Hang Baishao’; rather, ‘Qihua Lushuang’, a typical northern cultivar, was at the top (Supplementary Table S36). This result is a little unexpected but a very good evidence to prove the objectivity of the strategy integrating the LQO and MCDM. ‘Qihua Lushuang’ is an excellent cultivar that has nearly all the good index characteristics necessary for planting in Hangzhou, such as a high number of beautiful flowers, more than 90% of flowering plants, strong fragrance, short dormancy duration and strong heat resistance. This cultivar can be used directly in parks and gardens of the central subtropical region and can also be adopted as a superior female to create the new peony germplasm^60,62^.

‘Hang Baishao’ is also a very good peony, having nearly all the same advantages as ‘Qihua Lushuang’. It was ranked second only because the scores of several low-weight indices were lower, such as “Fragrance level (*C*_6_)” (Supplementary Table S35). Even so, this unique peony native to southern China should be promoted as a pioneer material in the plant landscape and flower border designs in the low-latitude regions^29,30^. Actually, in China, ‘Hang Baishao’ is the only herbaceous peony that has the centuries-old and large-scale plantation and production in the low latitudes, and thus has strong adaptability under the hot and humid climates. More importantly, this special peony has the precious low-CR trait for peony breeding in southern China^3^. Therefore, we have adopted this southern peony as the main material to study the CR trait and bud dormancy mechanism of herbaceous peonies during the last several years^7,25^.

‘Hong Jinqiu’, ‘Yanzi Xiangyang’, ‘Zifeng Chaoyang’ had the representatively early sprouting and flowering, while ‘Yangfei Chuyu’, ‘Taohua Feixue’ and ‘Liantai’ had the typically late sprouting and flowering (Supplementary Tables S3 and S12). Adopting these cultivars rationally in plant landscape design could prolong the entire duration of appreciating flowers. These cultivars likely have genes associated with typically early- or late-flowering traits and are regulated by the chilling duration, which is valuable for peony crossbreeding^12,19,76–79^. ‘Yangfei Chuyu’, ‘Taohua Feixue’ and ‘Meiju’ were ranked highly and graded as “I” level, meaning they were highly recommended; whule ‘Zhuguang’ and ‘Qiaoling’ were the two worst peonies among the 30 cultivars, and they performed poorly in most aspects (Supplementary Tables S35 and S36). Even so, they probably have special potential values as research materials with the high CR trait; thus, they could be used to comparatively study the difference between cultivars with representative low and high CR traits, and the mechanisms of herbaceous peony bud dormancy induction, transition and release^68,69,72^. ‘Guanyin Zuo’ ranked the first both in the bud number and ornamental duration of TBSL, thus this is an excellent cultivar to appreciate the beautiful TBSL (Supplementary Tables S2 and S3). The comments of other cultivars were showed detailedly in Supplementary Table S36.

In conclusion, the integration of accurate data collected from LQO and the reasonable construction of the MCDM system can maximize the dependability, objectivity and comprehensiveness of the final evaluation results (Fig. 5). On the other hand, more studies on resource introduction, evaluation and crossbreeding still need to be carried out to promote the “Southward Planting of Herbaceous Peony” in the Northern Hemisphere.

## Methods

### Introduction and cultivation

A total of 29 northern cultivars of herbaceous peony were introduced from Heze city of Shandong Province in autumn of 2011, and the special southern peony ‘Hang Baishao’ was introduced from Pan’an County of Zhejiang Province in autumn of 2012 (Supplementary Table S1). The underground crowns of 30 cultivars were sterilized and then cultivated in the field with good drainage and ventilation of the Perennial Flower Resources Garden of Zhejiang University in Hangzhou (E 118°21’-120°30’, N 29°11’-30°33’, subtropical monsoon climate, Zone 9 in Supplementary Fig. S1), Zhejiang Province. The half of mixed buds were kept above the soil surface, and the interval space between each two crowns was 80.00 cm. All the original bud numbers of 30 cultivars in autumn of 2011 or 2012 were showed in Supplementary Table S2. The side buds were never removed for keeping the natural bud generation and growth.

Field management and fertigation during six years were carried out based on the principle of low maintainability, in order to provide the valuable reference to the rough and extensive cultivation of herbaceous peony in city parks. Peony plants were irrigated thoroughly when the 5.00 cm depth of soil layer was dry continuously for more than ten days in the relatively wet and hot seasons (such as July to September), or never irrigated in the rainy seasons (such as March to June). Fertilizations were conducted for twice in each year, the first was made before bud sprouting in the middle February, and the second was done before blooming in the late March. Fungicides and pesticides were never used in order to acquire the natural performance of 30 cultivars and evaluate their resistant levels under various disease and pest stresses.

The climatic data of each hour and each day during six experimental years in Hangzhou can be searched out in the web link that is “https://www.wunderground.com/weather/cn/hangzhou”.

### LQO of the annual growth cycles of 30 cultivars over six years from 2012 to 2017

The annual growth cycles were observed and measured rigorously from 2012 to 2017 to collect sufficient information needed for the subsequent evaluation. Details of the observed indices are presented in the notes of Supplementary Tables S2–S16 and S19. Partial columns of some Supplementary Tables are highlighted with a yellow background, which indicates that the indices in these tables were adopted in the subsequent evaluation system. Heatmaps were used to show the global tendency of nine important indices over six years (Genesis 1.7.6, Institute for Genomics and Bioinformatics, AUT)^7^.

### Heat resistance determination under natural high temperatures

The extremely and continuously high temperatures occurred in July to August of 2013 in Hangzhou, which broke the 50-year historical record of high temperatures in this city (Supplementary Table S37 in the sheet 2 of Supplementary Dataset)^35–37^, and provided a good opportunity to study the heat resistance of the herbaceous peony planting in the subtropical region south of the Yangtze River. Therefore, heat injuries of leaves and stems were observed under natural high temperatures in early August 2013, which was the hottest period of this summer. The heat injury index (HII) was graded as the stay-green level of plants (0 to 4 levels) and was calculated as follows: HII of one cultivar = Σ (Grade value × Plant number of this grade) / (Value of the highest grade × Total number of the graded plants)^80,81^. The relative chlorophyll content (RCC) of leaves was measured directly by a SPAD-502 PLUS chlorophyll meter (Konica Minolta Sensing, Inc., Osaka, Japan), and the average value was calculated by the three RCC values of the leaf apex, middle and base, respectively^64,71^. Chlorophyll fluorescence characteristics of eight cultivars with significant differences in heat resistance were also observed by an Imaging-PAM chlorophyll fluorescence system (WALZ, Germany), and the parameters of *F*_o_, *F*_m_, PSα, *F*_v_/*F*_m_, *Φ*_PSα_, and *q*_P_ were measured in August 2013^64,80^.

### Heat resistance determination under artificial high temperatures

The semi-lethal temperatures (lethal temperature of 50%, LT_50_ for short) of leaves were measured for 30 peony cultivars in June 2013 before the forthcoming high temperature. Uninjured and green leaves were collected into constant temperature and humidity chambers (Climacell, MMM, Germany). The temperature gradients in the chambers were adjusted to 25, 30, 35, 40, 45, 50, 55 and 60 °C, and the relative humidity was 80%. Leaves were wrapped in plastic packaging bags and treated at each temperature for 24 h; then, they were cut into pieces and immersed in purified water. The electrical conductivity (EC) of the water with leaf fragments was measured by a STARTER 3 C conductivity meter (Ohaus Instrument (Shanghai) Co. Ltd, China) before and after the water was boiled. The relative electrical conductivity (REC, %) was calculated as follows: REC = (EC of un-boiled water / EC of boiled water) × 100%. The values of the treatment temperature and corresponding RECs were input into Origin 8.6 (OriginLab Corp., Northampton, MA) in pairs, and then the Slogistic1 curves and LT_50_ values were produced automatically^71,82,83^.

The HII, RCC and LT_50_ were regarded as three sub-indices to comprehensively evaluate the heat resistance of 30 herbaceous peony cultivars under natural and artificial temperatures in the hottest summer of Hangzhou. The relevant details are indicated in the notes of Supplementary Tables S17 and S34.

### Determination of disease and pest resistance, and final survival and non-degraded rates after a six-year cultivation

The degrees of injury from disease and pests were observed and visually graded in each summer of the six years^12^. The number of surviving and non-degraded plants was recorded after six years of cultivation in the summer of 2017. Details on the observations and calculations for these indices are elaborated in the notes of Supplementary Tables S20, S21 and S34.

### Crossbreeding between the southern and northern cultivars and subsequent observation

A total of 31 cross combinations of the southern peony ‘Hang Baishao’ and northern cultivars were designed based on the appropriate role determination of each cultivar in hybridization (Supplementary Tables S14 and S22). The anthers of male parents were collected on sunny and windless days and dried naturally by scattering pollen on filter paper in glass dishes. Female parents were emasculated beforehand and then pollinated artificially by dipping the dried pollen into stigmas. Then, pollinated flowers were covered by waxed paper bags^84–86^. Hybrid seeds were collected in mid-July after fruits became red, and they were immediately sown in seedbeds or pots.

### Data analysis

The experiments mentioned in this study were all performed in a randomized complete block design with three blocks and three replicates per block. Analysis of variance (ANOVA) was adopted to determine the statistical significance of the differences using SPSS 16.0 (significance levels are 0.05). All the photos or pictures were combined or arranged by the Microsoft Office PowerPoint 2016.

### Quantitative evaluation of the comprehensive performance by integrating the LQO and MCDM approaches

The AHP system, one of the MCDM approaches, was constructed to evaluate the comprehensive performance based on the six-year observations mentioned above. System construction included four steps as follows^12,14,15,31–33,49^.

### Step 1: hierarchy establishment and selection of evaluation indices

Three hierarchies were established as shown in Supplementary Table S23 and named as the target layer, constrained layer and index layer, respectively. The constrained layer included three categories named “Ornamental values (*B*_1_)”, “Resistances and dormancy duration (*B*_2_)” and “Abilities to propagate and create new germplasm (*B*_3_)”. A total of 17 representative indices were assigned to three categories, which were selected by specific study purposes, material traits and expert suggestions, and covered three main categories, i.e., ornamental, resistance and crossbreeding.

### Step 2: construction of judgement matrices and calculation of evaluation weights

The degrees of importance (scale values) were compared between two adjacent hierarchies, and then judgement matrices were constructed and consistencies were also examined (Supplementary Tables S24–S29). Scale values among different indices were input into the MATLAB programming language (version R2016a maci64; MathWorks, Natick, MA), and index weights were produced automatically (Supplementary Tables S30–S32). Then, the entire evaluation system was acquired with the integrated layers, indices and corresponding weights (Supplementary Table S33).

### Step 3: establishment of criteria for grading and scoring the 17 indices of the *C* layer

Based on the data from LQO in the nursery and laboratory, 17 indices were graded to five or three levels and scored from 20 to 100 points (Supplementary Table S34). Criteria for grading and scoring were strictly determined according to the six-year observation data, details of which are shown in Supplementary Tables S2–S22 and S34.

### Step 4: final scoring, calculating and ranking

The scoring for each index was regarded as a criterion and the calculation of a single score was determined for each index by weight values. A summary of each 17 single scores was calculated as the total score of one cultivar. The 30 total scores were ranked and then a ranking list was acquired. The final ranking of 30 cultivars was graded equally to five levels (six cultivars in each level), which could reflect the different recommendation degrees of 30 cultivars for using as a landscape plant in Hangzhou.

## Supplementary information

Dataset 1

## Data Availability

All data generated or analyzed during this study are included in this published article and its Supplementary Dataset (an Excel file).

## References

[1] Cohen M, Kamenetsky R, Din GY (2016). Herbaceous peony in warm climate: Modelling stem elongation and growers profit responses to dormancy conditions. Information Processing in Agriculture.

[2] Peng LP (2019). Modelling environmentally suitable areas for the potential introduction and cultivation of the emerging oil crop *Paeonia ostii* in China. Sci Rep.

[3] Zhuang WB, Cai BH, Gao ZH, Zhang Z (2016). Determination of chilling and heat requirements of 69 Japanese apricot cultivars. Eur. J. Agron..

[4] Guo RX, Li X, Luo GJ, Zhang TC, Lei JJ (2018). Investigation and taxonomy of wild *Fragaria* resources in Tibet, China. Genet. Resour. Crop Evol..

[5] Du YP (2014). Investigation and evaluation of the genus *Lilium* resources native to China. Genet. Resour. Crop Evol..

[6] Jia Y (2014). Collection and evaluation of *Primula* species of western Sichuan in China. Genet. Resour. Crop Evol..

[7] Zhang JP (2017). Mining and expression analysis of candidate genes involved in regulating the chilling requirement fulfillment of *Paeonia lactiflora* ‘Hang Baishao’. BMC Plant Biol..

[8] Li, Q. W. The Theory and Practice of Northward Plantation of Mei Flower—Commemorating Academician Chen Junyu. *Chinese Landscape**Architecture* **28**, 42–45 (in chinese) (2012).

[9] Di Lena B (2018). Impact of climate change on the possible expansion of almond cultivation area pole-ward: a case study of Abruzzo, Italy. J. Horticult. Sci. Biotechnol..

[10] Vitasse Y, Signarbieux C, Fu YH (2018). Global warming leads to more uniform spring phenology across elevations. Proc. Natl. Acad. Sci. USA.

[11] Zhang, J. P., Li, D. Q., Li, K. & Xia, Y. P. Reconsideration for the Southward Plantation of *Paeonia lactiflora*. *Chinese Landscape Architecture*. **32**, 91–95 (in chinese) (2016).

[12] Zhang MM, Huang H, Wang Q, Dai SL (2018). Cross Breeding New Cultivars of Early-flowering Multiflora *Chrysanthemum* Based on Mathematical Analysis. Hortscience.

[13] Ozdemir S, Sahin G (2018). Multi-criteria decision-making in the location selection for a solar PV power plant using AHP. Measurement.

[14] Wei X (2018). Development of a multi-criteria decision making model for evaluating the energy potential of Miscanthus germplasms for bioenergy production. Ind. Crop. Prod..

[15] Dragincic J, Korac N, Blagojevic B (2015). Group multi-criteria decision making (GMCDM) approach for selecting the most suitable table grape variety intended for organic viticulture. Comput. Electron. Agric..

[16] Zhao DQ, Wei MR, Shi M, Hao ZJ, Tao J (2017). Identification and comparative profiling of miRNAs in herbaceous peony (*Paeonia lactiflora* Pall.) with red/yellow bicoloured flowers. Sci Rep.

[17] Zhao DQ, Hao ZJ, Wang J, Tao J (2013). Effects of pH in irrigation water on plant growth and flower quality in herbaceous peony (*Paeonia lactiflora* Pall.). Sci. Hortic..

[18] Takahashi H (2014). The Gentio-Oligosaccharide Gentiobiose Functions in the Modulation of Bud Dormancy in the Herbaceous Perennial *Gentiana*. Plant Cell.

[19] Mouhu K (2013). The *Fragaria vesca* Homolog of Suppressor of overexpression of constans1 Represses Flowering and Promotes Vegetative Growth. Plant Cell.

[20] Yeo SM, Rhie YH, Lee SY, Jung HH, Kim KS (2012). Dormancy release and flowering of *Paeonia lactiflora* ‘Taebaek’ by natural cumulative chilling and GA_3_ treatment. Hortic. Environ. Biotechnol..

[21] Li XT, Liu P, Yang PP, Fan CZ, Sun XM (2018). Characterization of the glycerol-3-phosphate acyltransferase gene and its real-time expression under cold stress in *Paeonia lactiflora* Pall. PLoS One.

[22] Shen, C. Y., Tian, D. K., Zeng S. J. & Liao Z. L. Preliminary study on adaptation and performance of herbaceous peony (*Paeonia* L.) open-cultivated in south china. *Guangdong Agricultural Sciences*. 39, 39-42 (in chinese) (2012).

[23] Qin, K. J. & Li, J. Y. *Herbaceous Peony* (in chinese) 17, 69, 95, 99–100 (Shanghai Scientific & Technical Publishers, 2000).

[24] Yu, S. J., Yang, Y. Y. & Yu, S. X. *Herbaceous and Tree Peonies* (in chinese) 47, 96–97, 101, 132, 137–138 (China Agricultural Press, 2006).

[25] Zhang JP (2015). Transcriptomic Analysis of the Underground Renewal Buds during Dormancy Transition and Release in ‘Hangbaishao’ Peony (*Paeonia lactiflora*). PLoS One.

[26] Cheng, M. L., Lv, C. P. & Mo N. J. Research and Evaluation of *Paeonia Lactiflora* in Changsha. *Journal of Heilongjiang August First Land Reclamation University*. **20**, 32–35 (in chinese) (2008).

[27] Liu, L. Y. Studies on the Physiological and Bochemical Character of Heat and Humidity Tolerant *Paeonia lactiflora*. M.Sc. Thesis, Hunan Agricultural University (in chinese) (2008).

[28] Lv, C. P. & Liu, L. Y. Effects of high temperature on physiological and biochemical characteristics of *Paeonia lactiflora*. *Journal of Hunan Agricultural University* **34**, 664–667 (in chinese) (2008).

[29] Zhao DQ, Han CX, Zhou CH, Tao J (2015). Shade ameliorates high temperature-induced inhibition of growth in herbaceous peony (*Paeonia lactiflora*). Int J Agric Biol.

[30] Hao ZJ, Wei MR, Gong SJ, Zhao DQ, Tao J (2016). Transcriptome and digital gene expression analysis of herbaceous peony (*Paeonia lactiflora* Pall.) to screen thermo-tolerant related differently expressed genes. Genes Genom..

[31] Saaty RW (1987). The analytic hierarchy process - what it is and how it is used. Math. Model..

[32] Sanchez-Lozano JM, Fernandez-Martinez M (2016). Near-Earth object hazardous impact: A Multi-Criteria Decision Making approach. Sci Rep.

[33] Fang F, Qiao LL, Ni BJ, Cao JS, Yu HQ (2017). Quantitative evaluation on the characteristics of activated sludge granules and flocs using a fuzzy entropy-based approach. Sci Rep.

[34] Potgieter LJ (2018). Managing Urban Plant Invasions: a Multi-Criteria Prioritization Approach. Environ. Manage..

[35] Shu L (2016). Integrated studies of a regional ozone pollution synthetically affected by subtropical high and typhoon system in the Yangtze River Delta region, China. Atmos. Chem. Phys..

[36] Wang, Y., Liu, D. N. & Zhang, W. W. Characteristics of Hot Weather and Its Influence Factors from 1951 to 2015 in Hangzhou of Zhejiang Province. *Journal of Arid Meteorology*. **35**, 611–618 (in Chinese) (2017).

[37] Xie, Z. Q., Du, Y., Zeng, Y., Gao, P. & Miao, Q. Impact of urban clusters on spatial pattern of extreme high temperature events over Yangtze River Delta. *Chin Sci Bull* **62**, 233–244 (in Chinese) (2017).

[38] Evans LM (2014). Population genomics of *Populus trichocarpa* identifies signatures of selection and adaptive trait associations. Nature Genet..

[39] Daviere J-M, Achard P (2016). A Pivotal Role of DELLAs in Regulating Multiple Hormone Signals. Mol. Plant..

[40] Zhang QX (2012). The genome of *Prunus mume*. Nat. Commun..

[41] Zhang QX (2018). The genetic architecture of floral traits in the woody plant *Prunus mume*. Nat. Commun..

[42] Leeggangers HACF, Nijveen H, Bigas JN, Hilhorst HWM, Immink RGH (2017). Molecular Regulation of Temperature-Dependent Floral Induction in *Tulipa gesneriana*. Plant Physiol..

[43] Monsef HA-E, Hassan MAA, Shata S (2017). Using spatial data analysis for delineating existing mangroves stands and siting suitable locations for mangroves plantation. Comput. Electron. Agric..

[44] Cho S, Kim J, Heo E (2015). Application of fuzzy analytic hierarchy process to select the optimal heating facility for Korean horticulture and stockbreeding sectors. Renew. Sust. Energ. Rev..

[45] Garcia JL (2014). Multi-attribute evaluation and selection of sites for agricultural product warehouses based on an Analytic Hierarchy Process. Comput. Electron. Agric..

[46] Dehe B, Bamford D (2015). Development, test and comparison of two Multiple Criteria Decision Analysis (MCDA) models: A case of healthcare infrastructure location. Expert Syst. Appl..

[47] Kirubakaran B, Ilangkumaran M (2016). Selection of optimum maintenance strategy based on FAHP integrated with GRA-TOPSIS. Annals Of Operations Research.

[48] Emovon I, Norman RA, Murphy AJ (2018). Hybrid MCDM based methodology for selecting the optimum maintenance strategy for ship machinery systems. J. Intell. Manuf..

[49] Zheng Y, Meng TF, Bi XY, Lei JJ (2017). Investigation and evaluation of wild *Iris* resources in Liaoning Province, China. Genet. Resour. Crop Evol..

[50] Xue R (2019). A new method for soil health assessment based on Analytic Hierarchy Process and meta-analysis. Sci. Total Environ..

[51] Sarrou E (2017). Conventional breeding of Greek oregano (*Origanum vulgare* ssp. *hirtum*) and development of improved cultivars for yield potential and essential oil quality. Euphytica.

[52] Xing G (2017). Collection and evaluation of wild tulip (*Tulipa* spp.) resources in China. Genet. Resour. Crop Evol..

[53] Meng C (2014). An integrated simulation and AHP approach to vegetable grafting operation design. Comput. Electron. Agric..

[54] Yan ZG (2019). Phenotypic Characteristics and Fatty Acid Composition of Seeds from Different Herbaceous Peony Species Native to China. Chem. Biodivers..

[55] Li WX (2017). Nutritional evaluation of herbaceous peony (*Paeonia lactiflora* Pall.) petals. Emir. J. Food Agric..

[56] Luo JR (2017). Transcriptomic Analysis Reveals Transcription Factors Related to Leaf Anthocyanin Biosynthesis in *Paeonia qiui*. Molecules.

[57] Rhie YH, Jung HH, Kim KS (2012). Chilling requirement for breaking dormancy and flowering in *Paeonia lactiflora* ‘Taebaek’and ‘Mulsurae’. Hortic. Environ. Biotechnol..

[58] Park JH, Rhie YH, Lee SY, Kim KS (2015). Pre-chilling promotes flowering in *Paeonia lactiflora* ‘Taebaek’without flower bud abortion. Hortic. Environ. Biotechnol..

[59] Mornya PMP, Cheng F (2018). Effect of Combined Chilling and GA_3_ Treatment on Bud Abortion in Forced ‘Luoyanghong’ Tree Peony (*Paeonia suffruticosa* Andr.). Horticultural Plant Journal.

[60] Zhao DQ (2019). Overexpression of herbaceous peony *HSP70* confers high temperature tolerance. BMC Genomics.

[61] Zhao DQ, Hao Z, Tao J, Han C (2013). Silicon application enhances the mechanical strength of inflorescence stem in herbaceous peony (*Paeonia lactiflora* Pall.). Sci. Hortic..

[62] Tang YH, Zhao DQ, Meng JS, Tao J (2019). EGTA reduces the inflorescence stem mechanical strength of herbaceous peony by modifying secondary wall biosynthesis. Hortic. Res.-England.

[63] Zhao DQ (2019). Integration of Transcriptome, Proteome, and Metabolome Provides Insights into How Calcium Enhances the Mechanical Strength of Herbaceous Peony Inflorescence Stems. Cells.

[64] Xia X, Tang YH, Wei MR, Zhao DQ (2018). Effect of Paclobutrazol Application on Plant Photosynthetic Performance and Leaf Greenness of Herbaceous Peony. Horticulturae.

[65] Zhang YX, Wang YY, Gao XK, Liu CY, Gai SP (2018). Identification and characterization of microRNAs in tree peony during chilling induced dormancy release by high-throughput sequencing. Sci Rep.

[66] Zhang YX, Yu D, Liu CY, Gai SP (2018). Dynamic of carbohydrate metabolism and the related genes highlights PPP pathway activation during chilling induced bud dormancy release in tree peony (*Paeonia suffruticosa*). Sci. Hortic..

[67] Zhang YX (2016). MYC *cis*-Elements in *PsMPT* Promoter Is Involved in Chilling Response of *Paeonia suffruticosa*. PLoS One.

[68] Tylewicz S (2018). Photoperiodic control of seasonal growth is mediated by ABA acting on cell-cell communication. Science.

[69] Horvath DP, Anderson JV, Chao WS, Foley ME (2003). Knowing when to grow: signals regulating bud dormancy. Trends Plant Sci..

[70] Zhang YX, Zhang L, Gai SP, Liu CY, Lu S (2015). Cloning and expression analysis of the R2R3-*PsMYB1* gene associated with bud dormancy during chilling treatment in the tree peony (*Paeonia suffruticosa*). Plant Growth Regul..

[71] Li DQ, Zhang J, Zhang JP, Li K, Xia YP (2017). Green Period Characteristics and Foliar Cold Tolerance in 12 *Iris* Species and Cultivars in the Yangtze Delta, China. HortTechnology.

[72] Jansson S, Douglas CJ (2007). Populus: A model system for plant biology. Annu. Rev. Plant Biol..

[73] Yordanov YS, Ma C, Strauss SH, Busov VB (2014). EARLY *BUD-BREAK 1* (*EBB1*) is a regulator of release from seasonal dormancy in poplar trees. Proc. Natl. Acad. Sci. USA.

[74] Zhang YX (2015). Isolation and Characterization of a *SOC1-Like* Gene from Tree Peony (*Paeonia suffruticosa*). Plant Mol. Biol. Rep..

[75] Rinne PLH (2011). Chilling of Dormant Buds Hyperinduces *FLOWERING LOCUS T* and Recruits GA-Inducible 1,3-beta-Glucanases to Reopen Signal Conduits and Release Dormancy in *Populus*. Plant Cell.

[76] Hou XG (2018). Screening of Genes Related to Early and Late Flowering in Tree Peony Based on Bulked Segregant RNA Sequencing and Verification by Quantitative Real-Time PCR. Molecules.

[77] Lazaro A, Obeng-Hinneh E, Albani MC (2018). Extended Vernalization Regulates Inflorescence Fate in Arabis alpina by Stably Silencing *Perpetual flowering1*. Plant Physiol..

[78] Yang YY (2014). A Zinc Finger Protein Regulates Flowering Time and Abiotic Stress Tolerance in *Chrysanthemum* by Modulating Gibberellin Biosynthesis. Plant Cell.

[79] Wei Q (2017). Control of chrysanthemum flowering through integration with an aging pathway. Nat. Commun..

[80] Poudyal D, Rosenqvist E, Ottosen CO (2019). Phenotyping from lab to field - tomato lines screened for heat stress using F_v_/F_m_ maintain high fruit yield during thermal stress in the field. Funct. Plant Biol..

[81] Tian Z. G., Wang F., Zhang W. E. & Zhao, X. M. Effects of Heat Stress on Growth and Physiology of Marigold Cultivars. *Acta Horticulturae Sinica*. **38**, 1947–1954 (in Chinese) (2011).

[82] Liu B, Zhou H, Cao S, Xia YP, Arora R (2017). Comparative Physiology of Natural Deacclimation in Ten Azalea Cultivars. Hortscience.

[83] Fiebelkorn D, Horvath D, Rahman M (2018). Genome-wide association study for electrolyte leakage in rapeseed/canola (*Brassica napus* L.). Mol. Breed..

[84] de Souza EH (2017). Interspecific and intergeneric hybridization in Bromeliaceae and their relationships to breeding systems. Sci. Hortic..

[85] Andrieu E, Debussche M, Galloni M, Thompson JD (2007). The interplay of pollination, costs of reproduction and plant size in maternal fertility limitation in perennial Paeonia officinalis. Oecologia.

[86] Hao Q (2013). Crossability of American tree peony ‘High Noon’as seed parent with Japanese cultivars to breed superior cultivars. Euphytica.

